# Explosive Weapons Trauma Care Collective (EXTRACCT) Clinical Practice Guideline: Resuscitation of Pediatric Blast Injury Patient

**DOI:** 10.1002/wjs.70186

**Published:** 2025-12-21

**Authors:** Gavin Wooldridge, Francis Abantanga, Emmanuel Ameh, Vinay N. Kampalath, Paul Reavley, Philip C. Spinella, the EXTRACCT Clinical Practice Guidelines Collaboration

**Affiliations:** ^1^ Northwest and North Wales Pediatric Transport Service (NWTS) Warrington UK; ^2^ School of Medicine and Health Sciences University of Development Studies Tamale Ghana; ^3^ National Hospital Abuja Nigeria; ^4^ Department of Pediatrics, Perelman School of Medicine University of Pennsylvania Philadelphia Pennsylvania USA; ^5^ Bristol Royal Hospital for Children Bristol UK; ^6^ Department of Surgery and Critical Care Medicine, Trauma and Transfusion Medicine Research Center University of Pittsburgh Pittsburgh Pennsylvania USA

**Keywords:** assessment, blast injury, critical care, management, pediatrics, trauma

## Abstract

**Introduction:**

Children living in conflict or post‐conflict zones are frequently exposed to explosive injuries, with thousands killed and injured every year. The clinical practice guideline from the Explosive Weapons Trauma Care Collective (EXTRACCT) group provides a review of current best practice for the resuscitation of a child who has sustained a blast injury in low‐resource settings.

**Methods:**

An expert literature review of current practice was undertaken.

**Results:**

The guideline relates to the specific considerations of pediatric resuscitation of a child with a blast injury in low‐resource settings. It aims to provide guidance to all health care professionals working in resource‐constrained, secondary‐level healthcare contexts. It takes into consideration clinical decision‐making and treatment algorithms where resource availability is limited with respect to equipment and materials, subspecialist expertise, and critical care capabilities.

**Conclusion:**

The strength of the CPG recommendations is limited by a lack of data on pediatric blast victims. Future work is required, including establishing a blast injury victim registry and clinical trials on blast injury management strategies.

## Graphical abstract

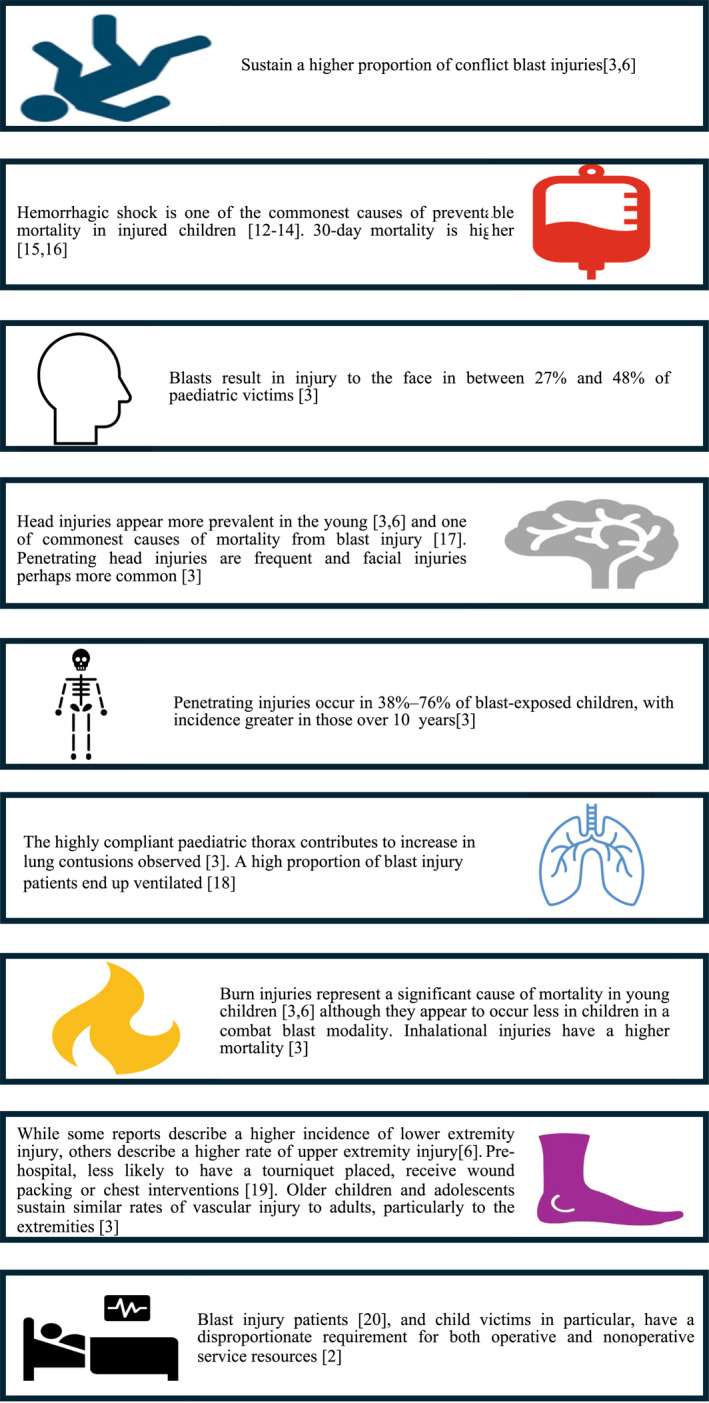

## 1 Objectives

The Resuscitation of Pediatric Blast Injury Patient Clinical Practice Guideline (CPG) encompasses the resuscitation and stabilization of a child who has sustained a blast injury. It relates to the specific considerations of pediatric resuscitation and aims to provide guidance to all health care professionals. It does not cover the detailed management of specific injury types (e.g., burns, amputation, traumatic brain injury, etc.). These can be found in the pediatric subsections of the relevant EXTRACCT CPGs. This CPG is intended as a concise and practical guide for all levels of secondary healthcare in a low‐resource setting. It could be used at multiple levels of care, from stabilization point to the emergency department of a tertiary hospital, but is designed with more resource‐limited environments in mind, such as district hospitals or casualty stabilization points. The recommendations are tiered to reflect resources available.

## 2 Background

Approximately one in six children worldwide lives in a conflict zone, with thousands killed and maimed each year [1]. Millions of children have been left permanently disabled over the last decade due to conflict [2], with many more affected indirectly. Children living in conflict or post‐conflict zones are frequently exposed to high‐order explosives [3], with most pediatric conflict deaths related to blast rather than ballistic injuries [4, 5, 6].

A blast injury describes any injury caused by the rapid pressure wave and associated fragments generated by an explosion [7]. The severity of injury depends upon many factors including the size and type of device (which can range from anti‐personnel mine [APM] to improvised explosive devices [IEDS]), the physical geography (outside or inside a building), and the victim (proximity to the device, their size, presence of personal protective equipment, and incidence of burns). The effects of blast are traditionally split into four or five theoretical categories [8]. However, this originated from predominantly open‐field Second World War data and does not account for the modern variations in explosive type and environment [9]. There is often an overlap in injury mechanisms, and children often have more than one body system affected.

- Primary Blast Injury. This is the effect of the blast pressure wave as it passes through the body depositing energy, particularly at the gas‐liquid interface
- Secondary Blast Injury. Blunt or penetrating trauma caused by fragments from the bomb itself or environmental debris
- Tertiary Blast Injury. The blast causes physical displacement of the body and impact with surrounding objects
- Quaternary blast injuries—Burns, inhalation, toxic or exacerbation of pre‐existing medical conditions
- (Quinary blast injury—Clinical consequences of post detonation environmental contaminants, e.g., sarin, anthrax)

Children are not a uniform demographic, and injury patterns and presentation will vary with age and context. For example, whilst blast injuries are more likely to kill younger children, older children have similar injury patterns to adults [3, 6]. The unique anatomy, physiology, size, and development of children would mean that the same explosive devices designed to wound an adult combatant can more easily kill a child.

Non‐standardized reporting means it is challenging to draw many conclusions related to epidemiology or injury patterns. Many critically ill children will die before reaching hospital [10]. Males appear to be the most commonly injured. Children aged 4–10 years appear most affected during conflict [3]. Mortality ranges from 8% to 11% [4, 5, 6, 11] depending upon context and definitions, with higher rates described from land mines [3].

**FIGURE 1:**
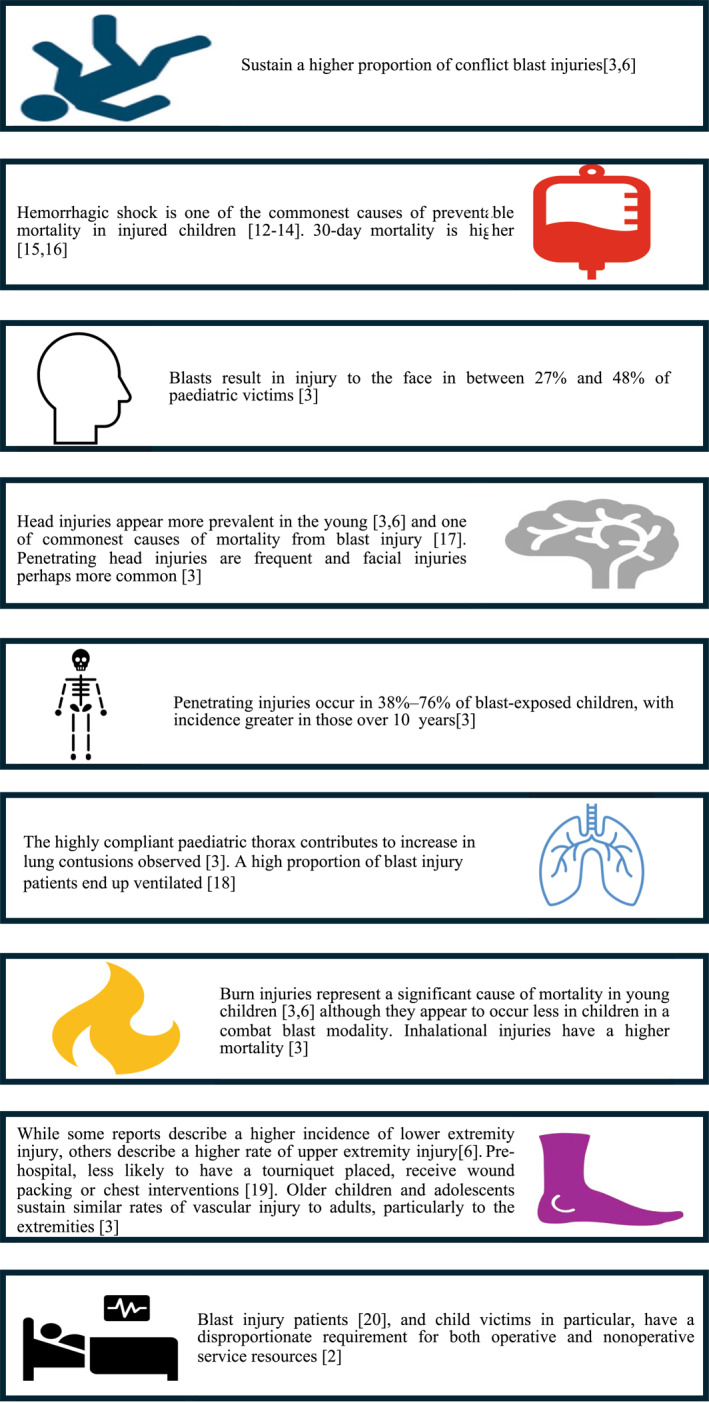
How pediatric blast injuries may differ from adults [2, 3, 6, 12, 13, 14, 15, 16, 17, 18, 19, 20].

Reported injury patterns are variable, but blast injuries in children appear to differ from those in adults, with specific sub‐populations at particular risk of worse outcomes from blast injury (Figure 1). These include those under the age of 5 years, those with associated burns, and children with head injuries [3, 6].

## 3 Review Method

A formal literature review was outside the scope of the CPG aims. Three recent systematic reviews on Pediatric Blast Injury were identified, references synthesized, and taken into account during CPG development [3, 6, 21]. No indication existed to repeat a review given prior work on this subject.

The CPG has been structured in accordance with the EXTRACCT CPG process [22].

### 3.1 Patient Evaluation—Pediatric Considerations in Resuscitation of Blast Injury Victim

Assessment and management of the pediatric blast injury patient proceeds in a systematic fashion, following the trauma primary survey. Children often sustain injuries to multiple body regions [23]; and a thorough examination including a full secondary survey is therefore required, with a high index of suspicion for further injury. Children may have been trapped under the rubble for extended periods, and consideration must be given to the presence and consequences of crush injuries.

A team‐based approach is best practice, incorporating features of effective teamwork, including closed‐loop communication, resource management, and shared decision‐making. Each health care member is allocated a role, and assessment and procedural activities occur simultaneously during patient resuscitation. Use of the World Health Organization (WHO) Interagency Integrated Triage Tool (WHO, ICRC, MSF) [24] and the WHO trauma resuscitation algorithm [25] is essential in saving lives. These allow the early recognition and prompt treatment (and referral if required) of the critically injured child, including surgery for rapid restoration of physiology as part of a damage control resuscitation approach.

**FIGURE 2:**
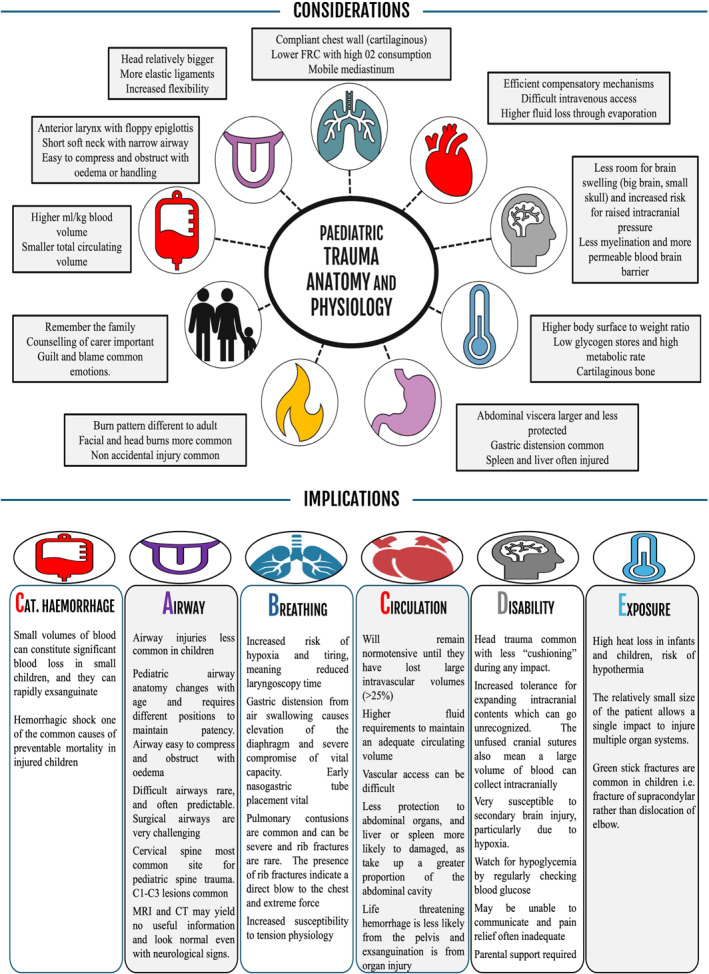
Pediatric trauma considerations and implications.

Below are specific considerations during the cABCDE assessment in secondary level healthcare related to the unique features of pediatric anatomy, physiology, and blast injuries (Table 1 and Figure 2). Key life‐threatening injuries and treatment goals are also addressed. The tiered approach reflects the resources available, both in terms of clinical expertise and equipment.

- Good
  ◦ Essential critical care achievable with minimal equipment and staff should be available to all critically ill children [50]
    – Level of treatment expected in WHO Level 1 facilities, with recommended level of monitoring, equipment, and medications [51, 52]
- Better
  ◦ Next level of critical care, requiring additional resources and clinical expertise
    – Level of treatment expected in WHO Level 2 facilities, with recommended level of monitoring, equipment, and medications [51, 52]
- Best
  ◦ Advanced level of critical care, with access to intensive care resources and staff.
    – Level of treatment expected in WHO Level 3 facilities, with recommended level of monitoring, equipment, and medications [51, 52]

**TABLE 1:** Specific considerations in pediatric CABCDE assessment.

Primary survey	Life‐threatening injuries to exclude/treatment goals	Tiered approach	Pediatric trauma resuscitation suggestions
C	*Assessment* Exclude active hemorrhage *Goal* To stop all bleeding	Good	Immediately stop any compressible bleeding. See individual EXTRACCT CPGs on extremity injury, amputation, hemorrhage, and damage control.
		Better	Minimize patient handling to preserve clot formation. Obtain early intravenous or intraosseous access. In life threatening bleeding, initiate balanced transfusion with whole blood or with blood components as soon as appropriate and dose to effect with careful monitoring to prevent over‐resuscitation. Limit use of crystalloids.^a^
		Best	15 mg/kg tranexamic acid if available, (max dose 1 g) [26] within 3 h, if possible, with continuous intravenous infusion (2 mg/kg/hr) for at least 8 h or until bleeding stops OR 25 mg/kg tranexamic acid (max dose 2 g) and no infusion [27].
A	*Assessment* Exclude airway obstruction or rupture *Goal* Ensure patent airway while protecting the c spine	Good	Gentle handling of airway. A two‐person technique may be preferable in those with suspicion of cervical spine injury. Consider age and development for appropriate airway positioning, with the goal of placing in the child in the “sniffing position”. If significant oral/facial bleeding, maintain open airway, regular suctioning, consider packing with epinephrine‐soaked gauze, and manage patient on their side.
		Better	Consider intubation if situation and resources allow. Use local intubation checklist, including implementation of difficult airway guidelines. In the presence of facial burns or significant facial trauma with oral bleeding, secure the airway early with endotracheal intubation and ventilation. Do not cut the endotracheal tube and review regularly to ensure not displaced/moved. Consider nebulized tranexamic acid for persisting oral/pulmonary bleeding [28]. See Airway EXTRACCT CPG
		Best	C‐spine immobilization should only be applied when the child is compliant or when unconscious. Cervical collars should not be used in the conscious child. Blocks and tape or manual inline stabilization (MILS) are appropriate but only if tolerated. C spine immobilization is likely to make airway management challenging. Management of the airway always takes precedence.
B	*Assessment* Exclude: Pneumothorax Massive hemothorax Flail chest pulmonary contusion *Goal* O_2_ sats > 94% and adequate ventilation^b^	Good	Assist ventilation if significant or insufficient work of breathing. Clinically exclude and treat pneumo/hemothoraces. See Thoracic Injury EXTRACCT CPG.
		Better	Placement of oro/naso gastric tube for gastric decompression. Place with caution in facial trauma using oral route. Chest X ray or lung ultrasound—Is there evidence of lung injury? (Blast lung seen as “butterfly” pattern on cxr)/pneumo/hemothorax. Primary Blast Lung Injury (PBLI). All those suspected of PBLI require supportive care and observation in a high dependency environment. Patients who are asymptomatic 6 h after exposure can be discharged from close medical observation [8].
		Best	All children require pre‐oxygenation and gentle bagging during induction of anesthesia. In the presence of significant bleeding from the lungs, use a high peep during bag valve mask ventilation and use nebulized tranexamic acid [29]. Aim for tidal volumes 6–8 mL/kg [30]. Start with a Positive End Expiratory Pressure (PEEP) of 5 and titrate as required
C	*Assessment* Exclude: Cardiac tamponade Shock state *Goal* Improving tissue perfusion and cardiovascular stability	Good	Aim for a palpable radial pulse in the initial stages of resuscitation and gradual improvement of pulse rate and signs perfusion. Use 5–10 mL/kg of blood^c^ [31, 32] (if available and ideally warm) or crystalloid to reverse shock and improve perfusion. If hemodynamically unstable from bleeding, warmed blood or fluid must be administered as rapidly as possible and titrated to clinical effect. Reassess perfusion post bolus using heart rate, respiratory rate, peripheral pulses, capillary refill, AVPU (Alert, Voice, Pain, Unresponsive) or Glasgow Coma Score (GCS), as well as listening to lung sounds and feeling for a liver edge.
		Better	Extended Focused Assessment with Sonography in Trauma (EFAST) if no computed tomography (CT) available—evidence of free fluid? Be aware of the limitations of FAST in pediatric trauma [33, 34].^e^ ^,^ ^f^ A focused cardiac point of care ultrasound may help to determine the need for ongoing fluid resuscitation. Evaluation of the IVC may help assess fluid status. Exclude tamponade (rare). Limit use of crystalloids, warmed whole blood is preferable if available. Bind pelvis if suspicion of injury, and ensure injured long limbs splinted. Dress wounds and review any tourniquets.
		Best	Blood products should be given as either whole blood or in a ratio of 1 RBC: 1 FFP: 1 platelets. If available, pH, potassium, and ionized calcium should be regularly monitored^d^. Avoid hypothermia, which can contribute to coagulopathy. As traumatic brain injury (TBI) and pre‐hospital hypoxia are common in the pediatric blast injury population, hypotension should be avoided when TBI is suspected and age‐appropriate normal mean arterial pressure maintained [35, 36, 37, 38]. Significant hypertension can aggravate hemorrhage, but this may be a compensatory response to increased intracranial pressure. Although hypotension secondary to hemorrhage should be treated with blood, there is a role for the use of vasoactive agents, such as epinephrine (adrenaline) [39], especially in TBI and spinal injury (see Supporting Information S1).
D	*Assessment* For CNS involvement *Goal* Normoglycemia, no worsening of traumatic brain injury, good pain relief	Good	Closely monitor blood sugar. Hypoglycemia correction 3 mL/kg 10% dextrose [40, 41]. Early pain relief essential and close parental support vital. Broad spectrum antibiotics in penetrating head injury. Facial and eye injuries common. See Ophthalmic EXTRACCT CPG
		Better	Monitor and ensure early treatment of seizures, consider smaller benzodiazepine doses if AVPU/GCS low, and poor perfusion. If features of raised intracranial pressure (abnormal neurology, bradycardia, hypertension), consider hypertonic saline 2–5 mL/kg of 3% saline over 10–20 min and head up 30° [38]. Use mannitol 0.25–1.5 g/kg if hypertonic saline not available. Mannitol is often used but has a limited pediatric evidence base, beware in hypotension.
		Best	High prevalence of traumatic brain injury means neuroprotection is vital and requires maintenance of an adequate cerebral perfusion pressure [29]. Prophylactic treatment of seizures in the presence of TBI with phenytoin or levetiracetam [38]. See TBI EXTRACCT CPG. If injuries are clearly nonsurvivable, ensure sufficient pain relief and family presence [42].
E	*Assessment* Extent of injuries *Goal* Normothermia, identification and treatment of additional injuries	Good	Examine by exposing the child gradually region by region. Determine area and extent of burn. Prevent and aggressively treat hypothermia. Use WHO Trauma Care Checklist [43]. Up to 50% of pediatric deaths occur following hospital discharge [44], so ensuring all aspects of the checklist are completed is vital.
		Better	Splint limbs early to reduce bleeding and achieve pain relief. Give intravenous antibiotics in the presence of all blast related wounds with due consideration to local resistance patterns and need for repeated surgery [45]. In malaria endemic areas, do a malaria rapid diagnostic test.
		Best	Check tetanus status and treat appropriately [46]. Vitamin K if signs of coagulopathy (phytomenadione IV 300 μg/kg, max 10 mg). Consider urinary catheter placement in shocked patient, those at risk of rhabdomyolysis or ventilated patients. Avoid if signs of urethral/pelvic trauma. See pelvic Injury EXTRACCT CPG. Consideration must be given to blood‐borne virus prophylaxis [47].

^a^ Low titer group O whole blood is safer than ABO specific whole blood since it will avoid the risk of a fatal hemolytic reaction if mismatched whole blood is given. In locations where ABO typing is standard, it is acceptable to also use ABO specific whole blood.

^b^ WHO guidance recommends aiming for oxygen saturations > 94% [48] but accept lower oxygen saturations (> 88%) if high ventilatory pressures or high oxygen requirements.

^c^ Practice varies depending upon setting (i.e., 5 mL/kg is widely used in the United Kingdom while 10 mL/kg is used in the United States) [31, 32].

^d^ Limitations include its low sensitivity for solid organ injury, its accuracy is highly operator dependent, and a CT scan is still often required to make a full diagnosis and determine the extent of injuries [33, 34].

^e^ If adequate laboratory and blood product capabilities are available, a source of fibrinogen such as cryoprecipitate or fibrinogen concentrates should be used for acquired hypofibrinogenemia or reduced fibrinogen function [49]. In higher‐resourced settings where viscoelastic are available, these can be performed to assist with guiding resuscitation with blood products or intravenous hemostatic adjuncts. However, such studies are frequently unavailable at civilian facilities in resource‐limited conflict and post‐conflict settings.

^f^ Hypocalcemia management. Give 0.1 mL/kg of 10% calcium chloride for every 20 mL/kg of blood products or if ionized calcium level is less than 1.0 mmol/L. Or 0.1 mmol/kg (0.5 mL/kg) of calcium gluconate could be used to correct the hypocalcemia instead but requires higher volumes to achieve normal calcium levels. Hyperkalemia management: Potassium levels can rise to dangerous levels quickly with multiple blood products. Use a bolus of 10 mL/kg of 10% Dextrose and 0.1 units/kg of short‐acting insulin to maintain potassium below 6 mmol/L.

Future CPGs will address the pre‐hospital care of patients. An awareness of the normal age‐related vital signs is paramount (Table 2).

**TABLE 2:** Normal pediatric vital signs. Accrued from a range of data sources [53, 54, 55, 56].^a^

Age	Weight male (KG)	Weight female (KG)	HR	RR	⇩SBP
Newborn	3.3	3.2	90–180	40–60	60
1 M	4.5	4.2	110–180	30–50	70
3 M	6.4	5.8	110–160	30–35	70
6 M	7.9	7.3	100–150	30–35	72
1 Year	9.6	8.9	80–160	25–30	72
2 Years	12.2	11.5	80–140	20–28	74
4 Years	16.3	16.1	80–120	20–26	78
6 Years	20.5	20.2	75–115	18–24	82
8 Years	25.4	25.3	70–110	18–22	86
10 Years	31.2	31.9	70–110	16–20	90
12 Years	39	39	60–110	16–20	90
14 Years	49	50	60–100	16–20	92

^a^ Weights are taken from a variety of sources. Numerous formulas exist to calculate weight, but all have variable accuracy. Use of a Broselow tape is recommended but may overestimate the weight of those in low‐ and middle‐income countries.

### 3.2 Pediatric Vital Signs and Monitoring

An appreciation of the normal range of vital signs in children is essential when managing a child with a blast injury. An accurate weight should be obtained as soon as possible. We recommend that all critically ill children or children at high risk of deterioration should have continuous monitoring of essential vital signs, if resources allow:

◦ Oxygen saturation level
◦ Respiratory rate
◦ Heart rate
◦ Blood pressure
◦ AVPU or Glasgow Coma Score
◦ Pupillary size and response
◦ Blood sugar

The frequency of monitoring and observation depend upon the severity of injury. The sicker the child, the more frequent vital signs need monitoring. Continuous monitoring during the initial resuscitation is preferred. If there is a clinical change or deterioration, it is important to go back through the primary survey to identify and treat any new or evolving life or limb threatening injuries. Close relatives/parents can be instructed to contribute to clinical surveillance and notify medical or nursing staff if problems arise.

The ability to record and follow trends of observations is more informative than single readings and will provide early warning to the child's possible deterioration. There are many examples of warning score systems [57] with few validated in the humanitarian sector [58]. Table 2 demonstrates age‐based observations that are generally considered within normal range, although there is significant variability [59]. The systolic blood pressure column indicates the 5th centile and therefore indicates hypotension. This can be calculated from = 65 + (age in years × 2). If a child's observation lies outside these ranges, then an explanation must be rapidly sought and promptly rectified.

### 3.3 Intubation of a Critically Injured Child

If resources allow, intubation may be required in a variety of circumstances. There is, however, rarely a need to rush to intubate with good quality bag mask ventilation often lifesaving. It is imperative that intravascular volume is replaced in a child with hemorrhagic shock before induction and intubation. Cardiac arrest can ensue from a combination of factors including reduced venous return compounded by positive pressure ventilation, a fall in systemic vascular resistance, and myocardial depression from anesthetic drugs. A blast wave can lead to altered physiology, with profound activation of the autonomic nervous system, including apnea, hypotension, and bradycardia [60]. “Resuscitate before you intubate” and continue to oxygenate and ventilate as best as possible.

Intubation should be considered in

◦ Acute airway issues, including obstruction or hemorrhage
◦ Severe respiratory distress, progressive hypoxemia, or ventilatory failure
◦ Cardiovascular compromise
◦ “Humanitarian issues”—for example, multiple surgical procedures are imminent/severe injuries and/or pain
◦ Reduced level of consciousness generally considered a GCS < 8.

Rapid sequence intubation (RSI) of a critically injured child, especially one that is hemodynamically unstable, is challenging and the presence of other team members and checklists is essential. Intubation in this situation requires consideration of the child's Airway, Breathing, and Circulation as follows:

#### 3.3.1 Airway

With the high incidence of facial trauma and perhaps unfamiliarity of pediatric laryngoscopy, the intubator needs to be wary of a difficult airway. Best practice is to vocalize their airway plan to the team and prepare equipment accordingly using a local checklist including anticipation of a difficult airway [61]. High peep may be required to facilitate views of the larynx in the presence of airway bleeding.

- A microcuffed endotracheal tube (ETT) is recommended if available (Table 3).
  ◦ ETT Length: Thick black depth mark at level of the vocal cords
  ◦ Oral = Age/2 + 12
  ◦ Nasal = Age/2 + 15
- Stylet
  ◦ 2.0 Fr Stylet ETT up to 5.0 mm
  ◦ 4.0 Fr Stylet ETT > 5.0 mm
- Bougie
  ◦ Size 5 ETT sizes < 3.5 mm
  ◦ Size 10 ETT sizes 4–5.5 mm
- Suction catheter
  ◦ Twice internal diameter of ETT (e.g., 8Fr for a 4.0 mm ETT)

**TABLE 3:** Endotracheal and supraglottic device sizes.

Uncuffed ETT size	
Term neonate	3.5
5 months	4.0
> 1 year	(Age/4) + 4
**Microcuff ETT size**	
< 3 kg	Consider uncuffed
> 3 kg–8 months	3.0 mm
8 months–2 years	3.5 mm
2–4 years	4.0 mm
4–6 years	4.5 mm
6–8 years	5.0 mm
**Supraglottic device (LMA or IGEL)**	
Size 1	< 5 kg
Size 1.5	5–10 kg
Size 2	10–20 kg
Size 2.5	20–30 kg
Size 3	30–60 kg

#### 3.3.2 Breathing

**FIGURE 3:**
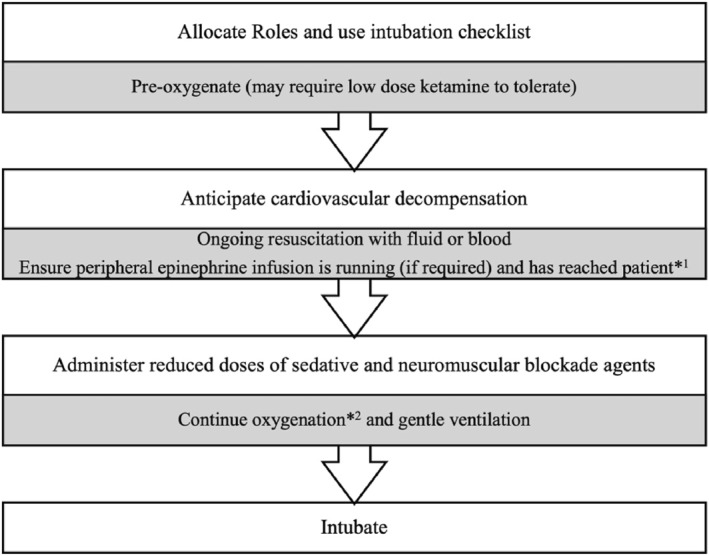
Rapid or delayed sequence intubation in the unstable cardiovascular pediatric blast injury patient. *1 Potentially evidenced by an increase in heart rate or blood pressure. *2 (including apneic oxygenation during intubation if possible).

All children and young people require pre‐oxygenation and gentle bagging during induction. On occasions, a small dose of sedative (e.g., 0.25–0.5 mg/kg IV ketamine) may be required to facilitate pre‐oxygenation (so‐called delayed sequence intubation [DSI]; Figure 3).

Consider the early placement of an oro or nasogastric tube (caution in facial injuries and suspected TBI, with orogastric the preferred route) and drain any pneumo/hemothoraces prior to intubation, or immediately after.

Primary blast lung injury (PBLI) can take different forms and appears to have both immediate and delayed effects [62]. PBLI is defined as an “acute lung injury within 12 h of blast exposure which is not due to penetrating or blunt injury” [63]. Apnea can occur immediately after the explosion, disruption of the air‐tissue interface can lead to alveolar hemorrhage, pulmonary contusions, air embolism [63], and pneumothoraces [9, 64]. Delayed effects include the progression of an inflammatory response [62]. Treatment is as outlined in Table 1, with attention directed to the immediate management of pulmonary hemorrhage, respiratory failure, and pneumo/hemothoraces. A low‐volume open lung protective lung approach is advocated for those patients with acute respiratory distress syndrome [62, 65].

#### 3.3.3 Circulation

Ideally the injured child should have 2 points of vascular access. Signs of poor perfusion or overt hypotension require ongoing resuscitation prior to induction of anesthesia. If whole blood is available, the use of low‐titer group O whole blood is preferable over ABO type‐specific if accurate ABO typing of the recipient cannot be ensured at a very high degree of certainty. There is no maximum dose of whole blood. The use of blood components in a 1:1:1 ratio should attempt to give each product in a balanced manner over time. While it is optimal to use RhD‐negative products for females before it is known, it is acceptable to use RhD‐positive blood products instead of deferring to non‐blood products if there are no RhD‐negative blood products available.

If, despite fluid/blood boluses of up to 20–40 mL/kg, poor perfusion, or hypotension persists, then it is not unreasonable to consider epinephrine (adrenaline) boluses and/or an infusion to improve hemodynamics, especially in the face of raised intracranial pressure. This can be commenced peripherally through an intravenous cannula or run central strength through an intraosseous cannula. The supplementary material contains how to calculate the peripheral epinephrine (adrenaline) infusion dose.

If worsening oxygenation precludes further cardiovascular stabilization, then doses of anesthetic agents need to be adjusted based on the child's hemodynamic status. Pediatric blast injury patients are likely to be cardiovascularly unstable and should receive lower doses of anesthetic agents for intubation (Table 4). It is important that as a team, you also consider other possible causes for the shocked status.

**TABLE 4:** Sedative agents.

Agent	Intubation dose	Dose in unstable patient
Fentanyl	1–5 mcg/kg	1–2 mcg/kg
Ketamine	1–2 mg/kg	0.5–2 mg/kg
Propofol	2–5 mg/kg	Avoid

As an example, for a child in hypovolemic shock, a suggested RSI regimen would be:

- Ketamine 0.5‐mg–1 mg/kg (can titrate to effect)
- +/− Fentanyl 1 mcg/kg
- Neuromuscular blockade (depending upon local preference and availability ‐be aware of side effects, onset, and duration of action), one of:
  ◦ Suxamethonium 1 mg/kg
  ◦ Rocuronium 1 mg/kg
  ◦ Atracurium 0.5 mg/kg

## 4 Pediatric Pain Relief

Pain is frequently under‐recognized and under‐treated in children [66]. An age and patient appropriate tool should be used to assess, score, and record pain. Interventions should be undertaken early and then reassessed.

Pain has physiological effects which are important to manage, even if the patient is unable to communicate their analgesic requirements. The triage tool below (Table 5) uses physiological signs and may serve as a useful guide for clinicians trying to determine analgesia requirements [42]. It has not yet been validated. Treat for severe pain if any major injury indicators are present. The presence of moderate injury indicators (without major injury signs) requires commencing analgesia for moderate pain. If only minor injury indicators are present, anticipate mild pain. A severe pain treatment algorithm can be found in the supplementary material.

**TABLE 5:** Physiological sign pain triage tool.

Injury indicators	Minor	Moderate	Major
Breathing	Normal or slightly faster breathing	Labored or rapid breathing	Absent or severely labored breathing
Circulation	No significant bleeding	Minor to moderate bleeding	Major bleeding or signs of hypotension
Consciousness	Conscious and alert	Altered mental state but not unresponsive	Unresponsive or significantly altered mental status
Injuries	Superficial or minor visible injuries	Visible injuries that are more than superficial	Multiple injuries in different body regions
LIKELY PAIN SEVERITY	MILD	MODERATE	SEVERE

### 4.1 Pediatric Traumatic Brain Injury

As mentioned previously, traumatic brain injuries appear common in pediatric blast injuries and are associated with worse outcomes. Good basic critical care is therefore paramount throughout the child's prehospital and emergency management to improve morbidity and mortality. A key component to remember is that cerebral perfusion pressure (CPP) is dependent on mean arterial pressure (MAP) and intracranial pressure (ICP), CPP (mmHg) = MAP − ICP. In order to prevent secondary cerebral injury, MAP should be maintained to target an estimated minimum CPP assuming that ICP is raised. The target MAP varies with age (Box 1).

**BOX 1 Neuroprotective measures and blood pressure targets.**

1

- Avoiding hypoxia, aim O_2_ saturations > 94%
- Maintenance of normocapnia, aim PaCO_2_ 35–40 mmHg.
- Maintenance of normotension

**Table:** 

MAP targets (mmHg)	
0–2 years	> 40
2–10 years	> 50
> 10 years	> 60
Systolic BP ranges (50th centile [67])	
0–1 year	75–95 mmHg
1–5 years	95–100 mmHg
5–12 years	100–110 mmHg
> 12 years	> 120 mmHg

- Aim Hb > 7 g/dL
- Maintenance of normoglycemia
- Maintenance of normothermia (< 38°C)
- Avoidance of seizures
- Neutral head positioning, remove any collar, loosen tube ties, and head of bed elevated 30° [36]

Hypotension should be actively avoided, as even a single episode of hypotension leads to worse outcomes in traumatic brain injury [35]. Given the high likelihood of TBI in blast injury, and the demonstrated poor outcomes with a single episode of hypotension in the brain‐injured child, permissive hypotension should not be employed in the unconscious child.

All factors that contribute to raised intracranial pressure, cerebral edema, and cerebral ischemia should be minimized (Box 1).

In the absence of radiology, a depressed conscious level is due to traumatic brain injury if other immediate causes, for example, shock or hypoglycemia, are excluded. An ocular point of care ultrasound and measurement of the optic nerve diameter can reveal intracranial hypertension [68]. Unequal pupils may suggest impending herniation. A blood sugar should always be checked. A 10% dextrose infusion may be required as part of their maintenance fluids to prevent further hypoglycemia. Be aware that even with a normal CT, a decreased conscious level may be related to blast traumatic brain injury or prolonged hypoxia/ischemia prior to arrival at the health facility. Cerebrovascular vasospasm may also occur in blast induced traumatic brain injury [69]. That being the case, neuroprotective measures are required. Seizures should be anticipated and emergency medication available to treat promptly.

### 4.2 Pediatric Transport Considerations

**FIGURE 4:**
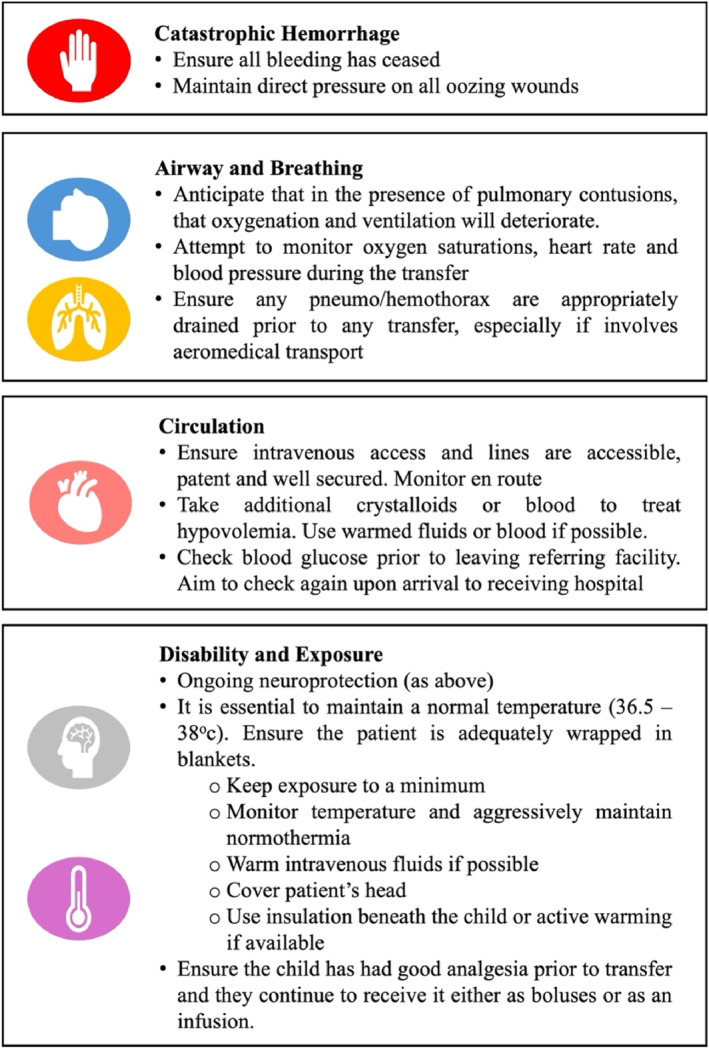
Transport consideration for a pediatric blast injury patient.

Prior to a transfer, there are specific considerations for a pediatric blast injury patient (Figure 4). Refer to a local transport checklist and the EXTRACCT Transport of Critically Injured Casualties CPG.

## 5 Data Collection and Quality Improvement

Clinical practice guidelines (CPG) serve numerous functions including: (i) standardization of care and adherence to evidence‐based care processes; (ii) improvement in clinical performance and care quality; and (iii) longitudinal monitoring of patient outcomes. Ongoing data collection of performance measures (including demographics of those affected) and desired outcomes in all pediatric patients who have suffered a blast injury is therefore essential to aid quality improvement [70]. A casualty data form for low‐resource conflict settings has been developed by EXTRACCT to support such efforts.

### 5.1 Conclusion

This CPG presents an overview of the resuscitation and stabilization of a child with a blast injury in low‐resource settings. The strength of evidence for these recommendations is limited by the lack of standardized data on pediatric blast injury victims and the difficulties in translating lessons in pediatric trauma management into health systems of low‐ and middle‐income countries. Future work should include the development of a pediatric blast injury victim registry and research trials on management strategies for blast injuries to strengthen available evidence beyond the descriptive retrospective reports currently available.

## Author Contributions

**Gavin Wooldridge:** conceptualization, writing – original draft, writing – review and editing, visualization. **Francis Abantanga:** writing – review and editing. **Emmanuel Ameh:** writing – review and editing. **Vinay N. Kampalath:** writing – review and editing. **Paul Reavley:** writing – review and editing. **Philip C. Spinella:** writing – review and editing.

## Ethics Statement

Ethics approval was not required as all data are in the public domain.

## Conflicts of Interest

The authors declare no conflicts of interest.

## Supporting information


Supporting Information S1
