# Supplementary material for: Explosive Weapons Trauma Care Collective (EXTRACCT) Clinical Practice Guideline: Resuscitation of Pediatric Blast Injury Patient
Source: World J Surg. 2025 Dec 21;50(1):162–76. doi: 10.1002/wjs.70186 (PMC12831528; doi:10.1002/wjs.70186)
Supplement: Supplementary file 1 — Supporting Information S1 [file WJS-50-162-s001.docx]

**Table of Contents for Supplements**

- Supplement 1a Epinephrine (adrenaline) infusion and bolus doses
- Supplement 1b Norepinephrine (noradrenaline) infusion
- Supplement 2 Intravenous sedation for ventilated patients
- Supplement 3 Pediatric fluid management
- Supplement 4 Severe pain management

**Supplement 1a – Epinephrine (adrenaline) infusion and bolus doses**

- It is recommended that a vasoactive infusion is always delivered via a dedicated peripheral line and that the line includes a 3-way tap
- How to calculate peripheral epinephrine (adrenaline) infusion dose [72]
  - Amount of 1:1000 (1mg/ml) Epinephrine (Adrenaline) = 0.3 x weight of patient =mg of epinephrine (adrenaline)
  - Add this (1mg=1ml) to 500ml 0.9% sodium chloride
- Worked example
  - If patient 14kg - 0.3 x 14kg = 4.2mg
  - Volume required of epinephrine (adrenaline) 1:1000 (1mg/ml) required = 4.2ml
  - Add 4.2ml of epinephrine (adrenaline) 1:1000 to 500ml 0/9% sodium chloride
  - Maximum peripheral epinephrine (adrenaline) concentration 16mg in 500ml
- Worked example to calculate dose from infusion rate, patient weight 16kg
  - Dose = 0.3 mg x 16 kg = 4.8 mg 4.8 ml added to 500 mL bag 0.9% sodium chloride
  - Rate running = 12 mL / hr
  - Step 1: Total dose = 4.8 mg x 1000 = 4,800 micrograms
  - Step 2: 4,800 micrograms ÷ 16 kg = 300 micrograms / kg
  - Step 3: 300 micrograms / kg ÷ 60 mins = 5
  - Step 4: 5 ÷ 500 mL = 0.01
  - Step 5: 0.01 x 12 mL = 0.12 micrograms / kg /min
- Alternatively, a bolus of dilute epinephrine (adrenaline) can be used
  - Take 0.1 mL/kg (10 micrograms/kg) from minijet syringe 1:10,000 epinephrine (adrenaline) (using a 3-way tap). Make this up to 10 ml with 0.9% sodium chloride (MAX: 1mg in 10ml i.e. neat).
  - Use 0.5-2 ml bolus if ↓BP at induction of anesthesia

Peripheral epinephrine (adrenaline) infusion chart

| PATIENT WEIGHT | MG TO ADD TO 500ML BAG | RATE (ML/HR) = 0.05MCG/KG/MIN | RATE (ML/HR) = 0.1MCG/KG/MIN | RATE (ML/HR) = 0.2MCG/KG/MIN | RATE (ML/HR) = 0.5MCG/KG/MIN |
| --- | --- | --- | --- | --- | --- |
| 3 | 0.9 | 5 | 10 | 20 | 50 |
| 5 | 1.5 | 5 | 10 | 20 | 50 |
| 10 | 3 | 5 | 10 | 20 | 50 |
| 20 | 6 | 5 | 10 | 20 | 50 |
| 30 | 8 | 5.6 | 11.3 | 22.5 | 56 |
| 40 | 8 | 7.5 | 15 | 30 | 75 |
| 50 | 8 | 9.4 | 18.8 | 37.5 | 94 |
| 60 | 8 | 11.3 | 22.5 | 45 | 113 |

**Supplement 1b – Norepinephrine (noradrenaline) infusion [71]**

- Amount of norepinephrine (noradrenaline) to add to 500 ml bag 0.9% sodium chloride = 0.3 mg x weight (kg)
- Maximum concentration = 8 mg in 500 ml. (Patients >100kg using dosing for 100kg)
- Dose: 0.05-1.5 micrograms/kg/min via peripheral line (for rate ml/hr see table below)

Peripheral norepinephrine (noradrenaline) infusion chart

| PATIENT WEIGHT (KG) | AMOUNT (MG) TO ADD TO 500ML BAG | RATE (ML/HR) = 0.05MCG/KG/MIN | RATE (ML/HR) = 0.1MCG/KG/MIN | RATE (ML/HR) = 0.2MCG/KG/MIN | RATE (ML/HR) = 0.5MCG/KG/MIN |
| --- | --- | --- | --- | --- | --- |
| 3 | 0.9 | 5 | 10 | 20 | 50 |
| 5 | 1.5 | 5 | 10 | 20 | 50 |
| 10 | 3 | 5 | 10 | 20 | 50 |
| 20 | 6 | 5 | 10 | 20 | 50 |
| 30 | 8 | 5.6 | 11.3 | 22.5 | 50 |
| 40 | 8 | 7.5 | 15 | 30 | 56 |
| 50 | 8 | 9.4 | 18.8 | 37.5 | 94 |
| 60 | 8 | 11.3 | 22.5 | 45 | 113 |

**Supplement 2 - Intravenous sedation for ventilated patients [71]**

**MORPHINE INFUSION**

- Weight in mg, made up to 50mls with 5% glucose or 0.9% sodium chloride
- 1ml/hr = 20mcg/kg/hr
- Range: 5 – 60mcg/kg/hr
- Maximum dose 50mg morphine in 50mls

Worked Example:

- If 10kg patient
- 10mg in 50mls 0.9 saline = 10000 mcg / 50 = 200mcg/ml
- 1ml/hr = 20mcg/kg/hr

How to calculate infusion rate (ml/hr):

- What you want to give (20mcg/kg/hr) divided by what you have (1000mcg/ml) = rate/hr

Calculation of morphine infusion

| PATIENT WEIGHT (KG) | AMOUNT (MG) TO ADD 50MLS | RATE (ML/HR) = 20mcg/kg/hr | RATE (ML/HR) = 40mcg/kg/hr |
| --- | --- | --- | --- |
| 3 | 3 | 1 | 2 |
| 5 | 5 | 1 | 2 |
| 10 | 10 | 1 | 2 |
| 20 | 20 | 1 | 2 |
| 30 | 30 | 1 | 2 |
| 40 | 40 | 1 | 2 |
| 50 | 50 | 1 | 2 |

MORPHINE BOLUS 50 – 100 MCG/KG

- Boluses of morphine are required to achieve effective plasma concentrations. If more than three boluses are required in one hour, increase the background rate by 20%. Occasional patients may need up to 60-80 mcg/kg/hr. Consider fentanyl in those who are resistant to morphine. Use lower doses for patients in hepatic or renal failure.

**Intravenous morphine dosing in ventilated patients > 50kg**

- 50mg in 50ml (1mg/ml) or 100mg in 50ml (2mg/ml)
- Bolus: 0.5mg - 2mg IV
- Range: 0 – 10mg/hr (usual 0.5 – 2.5mg), titrate to effect

**MIDAZOLAM INFUSION**

- Weight x 3, made up to 50mls with 5% glucose or 0.9% sodium chloride
- 1ml/hr = 60mcg/kg/hr
- Range: 60 – 300 mcg/kg/hr
- Max dose: 250mg in 50mls

How to calculate the infusion rate (ml/hr):

- Midazolam: What you want to give (1mcg/kg/min) x60 divided by what you have (1000mcg/ml) = rate/hr

Calculation of midazolam infusion

| PATIENT WEIGHT (KG) | AMOUNT (MG) TO ADD 50MLS | RATE (ML/HR) = 60mcg/kg/hr | RATE (ML/HR) = 120mcg/kg/hr |
| --- | --- | --- | --- |
| 3 | 9 | 1 | 2 |
| 5 | 15 | 1 | 2 |
| 10 | 30 | 1 | 2 |
| 20 | 60 | 1 | 2 |
| 30 | 90 | 1 | 2 |
| 40 | 120 | 1 | 2 |
| 50 | 150 | 1 | 2 |

MIDAZOLAM BOLUS 50 – 200 MCG/KG

- Use lower doses in those with hepatic or renal failure and in patients with hemodynamic instability

**Intravenous midazolam dosing in ventilated patients > 50kg**

- 50mg in 50ml (1mg/ml) or 100mg in 50ml (2mg/ml)
- Bolus: 0.5mg to 2.5mg
- Range 0 – 10mg/hr, usual 0.5 – 2.5mg, titrate to effect

Additional sedative agents

| AGENT | INFUSION DOSE RANGE |
| --- | --- |
| Fentanyl | 1 – 10mcg/kg/hr |
| Ketamine | 10 – 45mcg/kg/min |
| Propofol | 1 – 4mg/kg/hr |

**Supplement 3 – Pediatric Fluid management**

- For resuscitation, the preferred fluid type is sodium chloride 0.9% (with glucose 5% +/- potassium for maintenance fluid) or similar isotonic crystalloids such as plasmalyte or Hartmanns solution. Ideally blood should be used in an acute trauma situation with active bleeding.
- A fluid bolus should be given as quickly as possible, and the patient’s clinical response evaluated.
- For maintenance fluid, sodium chloride 0.9% with glucose 5% +/- potassium should be used depending upon the age of the patient and electrolytes.
- Full maintenance fluid rates can be calculated using the table below or other available guidelines [41][72]. They do not take into account ongoing losses nor prior fluid deficits:

Daily fluid requirements

| **Weight (kg)** | **Full maintenance ml/day** | **ml/hr** |
| --- | --- | --- |
| <10 | 100mls/kg | 4 |
| 10 - 20 | 50mls/kg | 2 |
| >20 | 20mls/kg (2400ml max) | 1 |

For a neonate fluids requirements are as follows:

Neonatal daily fluid requirement

| Day 1 | 60ml/kg/day |
| --- | --- |
| Day 2 | 80ml/kg/day |
| Day 3 | 90ml/kg/day |
| Day 4  Day 5 - 28 | 100ml/kg/day  120mls/kg day |

**Example calculations**

- An infant weighs 5 kg. What is the required amount of fluid per hour?
  - 5 kg x 100 mL/kg = 500 mL
  - Fluid rate of infusion per hour = 500/24
  - = 20.8ml/hr
- What is the fluid requirement of child weighing 33 Kg?
  - 100mls/kg for the first 10kg = 1000mls
  - 50mls/kg for the second 10kg = 500mls
  - 20mls/kg for all additional Kg (x 13)= 260 mls
  - Total = 1860mls
  - Rate = 1860/24 = 77.5 mls/hr
- What is the hourly fluid rate for a 3-day old baby?
  - 3 x 90mls/kg/day
  - =180mls/ day
  - 180/24 = 7.5mls/hr

However, most sick children, including trauma and post operative patients, will retain water as they secrete less anti-diuretic hormone. They, therefore, often require significantly less than full maintenance fluids.

- Calculate 80% maintenance for a 25 kg child post operative patient:
  - 100mls x 10kg = 1000ml
  - 50mls x 10 kg = 500ml
  - 20mls x 5 = 100mls
  - = 1600mls/day at full (100%) maintenance
  - 80% maintenance = 1600 x 0.8 = 1280mls/day = **53ml/hr**

**Supplement 4 – Severe pain management**

- Avoid non-steroidal active inflammatories (NSAIDS) in active bleeding, children < 6 months and those with suspected renal dysfunction (including those with crush injuries)

Figure 4 [46]. Used with permission from the Quick Guide: Emergency Pain Management for Injured Children. Paediatric Blast Injury Partnership, United Kingdom

^
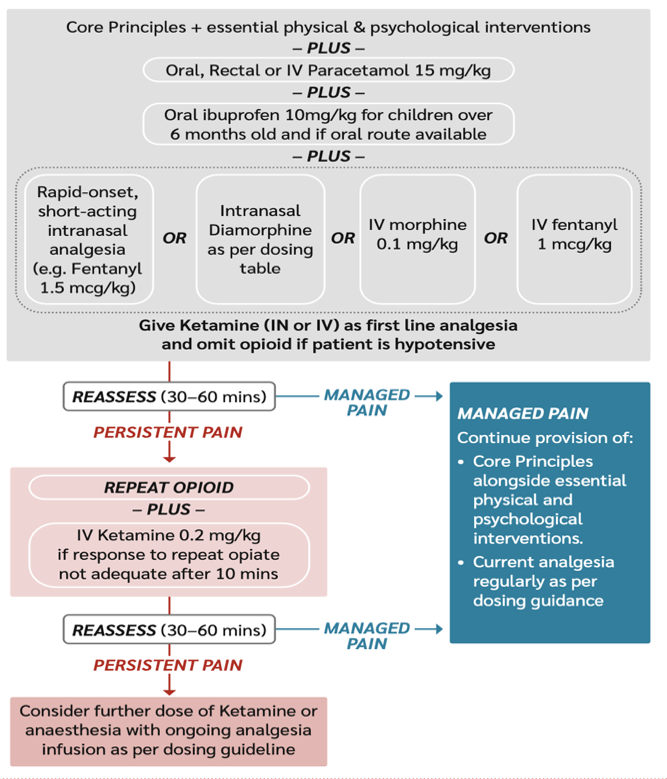
^
